# Atypical Presentation of Mpox as Acute Tonsillitis: A Case Report from the United Arab Emirates

**DOI:** 10.7759/cureus.113748

**Published:** 2026-07-31

**Authors:** Sara O Omer, Mortada Sayedalhassan Yasen Osman, Ahmad Younes, Tamadur Maweya Abdelbagi Albasheer, Sunil Kumar Shivarame Gowda

**Affiliations:** 1 Internal Medicine, Aldhafra Hospitals, Abu Dhabi Health Services Company, Pure Health Group, Abu Dhabi, ARE; 2 Emergency Medicine, Aldhafra Hospitals, Abu Dhabi Health Services Company, Pure Health Group, Abu Dhabi, ARE

**Keywords:** atypical presentation, case report, cervical lymphadenopathy, monkeypox, mpox, odynophagia, oropharyngeal infection, tonsillitis, united arab emirates

## Abstract

Mpox typically presents with a prodrome of fever and lymphadenopathy followed by a characteristic vesiculopustular rash. Mpox may present with atypical manifestations, including isolated oropharyngeal, anogenital, and mucosal involvement preceding cutaneous lesions. Recognition of these atypical phenotypes is essential to avoid diagnostic delay and limit secondary transmission, particularly in regions such as the United Arab Emirates and the wider Gulf where published descriptions of atypical presentations remain limited.

A previously healthy 28-year-old man in the United Arab Emirates presented with a two-day history of severe sore throat and painful swallowing that prevented oral intake. Examination revealed bilateral erythematous palatine tonsils with cryptic exudate, an edematous uvula, and pharyngeal erythema. There was no stridor or respiratory distress. Inflammatory markers were elevated (WBC count 14.0 × 10⁹/L; CRP 102 mg/L). Computed tomography of the neck demonstrated bulky, heterogeneous palatine tonsils with significant narrowing of the oropharyngeal lumen and reactive cervical lymphadenopathy; the radiologist’s impression raised the possibility of evolving abscess. A working diagnosis of acute bacterial tonsillitis was made, and intravenous ceftriaxone and dexamethasone were commenced. On hospital day 2, mild non-specific cutaneous lesions appeared, progressing on day 3 to a characteristic vesiculopustular eruption involving the face, trunk, palms, and genital area. Throat culture, finalised on day 4, yielded normal upper respiratory tract flora with no beta-haemolytic streptococci. Real-time PCR on a skin lesion swab confirmed Mpox virus infection (cycle threshold 12). The patient was isolated, transferred to a designated facility on hospital day 9, and discharged home on 11 January 2026 after a total of 27 days from initial admission. A structured post-discharge telephone follow-up on 12 January 2026 confirmed sustained clinical recovery with full lesion resolution and no recurrent symptoms.

Mpox can present initially as severe pharyngotonsillitis with significant oropharyngeal luminal narrowing, mimicking acute bacterial infection and preceding the appearance of skin lesions by several days, even in young, immunocompetent patients without recognised epidemiological risk factors. Clinicians should consider Mpox in patients with severe pharyngotonsillitis, particularly when bacterial cultures are negative or when even subtle cutaneous changes appear during admission, to enable timely isolation and appropriate management.

## Introduction

Mpox is a zoonotic orthopoxvirus infection that has historically been confined to endemic regions of Central and West Africa. Since May 2022, sustained human-to-human transmission has driven a global outbreak affecting more than 100 countries, prompting the World Health Organization to declare a Public Health Emergency of International Concern in 2022 and again in 2024 following the emergence of clade Ib in central Africa [1,2]. In the United Arab Emirates and the wider Gulf region, sporadic cases have been reported, predominantly linked to international travel [3]; published descriptions of locally relevant atypical clinical presentations remain limited. The classical clinical course consists of a febrile prodrome with lymphadenopathy followed within one to four days by a centrifugal vesiculopustular eruption that progresses through well-defined stages [4]. The 2022 outbreak, however, has been characterized by frequent atypical presentations. Mucosal involvement, including proctitis, urethritis, pharyngitis, and tonsillitis [5, 6], has been described, often preceding or occurring without the classical rash distribution. Severe oropharyngeal lesions causing odynophagia, dysphagia, and significant local inflammation have been documented in case series of clade IIb infection [7]. Such atypical presentations create a real risk of diagnostic delay. Patients may be admitted under alternative working diagnoses, most commonly acute bacterial tonsillitis or peritonsillar infection, and treated with empirical antibacterial therapy and adjunctive corticosteroids before Mpox is considered. This carries implications both for individual patient care and for infection control, as undiagnosed cases may expose healthcare workers and other patients before appropriate isolation is implemented. We report a case of laboratory-confirmed Mpox in a previously healthy, young man in the United Arab Emirates who presented with severe acute tonsillitis and CT evidence of significant oropharyngeal luminal narrowing, in whom the characteristic cutaneous eruption developed only after admission and after initial empirical antibacterial therapy. This case report was prepared in accordance with the CARE (CAse REport) Guidelines [2]. We aim to highlight the clinical features that should prompt consideration of Mpox in patients presenting with severe pharyngotonsillitis, including those without recognized epidemiological risk factors, and to discuss the practical infection-control implications of delayed recognition. To our knowledge, published reports from the United Arab Emirates describing Mpox presenting initially as severe acute tonsillitis with CT-confirmed oropharyngeal narrowing and without clinical airway compromise remain limited.

## Case presentation

A 28-year-old previously healthy man presented to the emergency department of a regional hospital in the United Arab Emirates with a two-day history of severe sore throat, progressive odynophagia, and inability to tolerate oral intake. He reported no fever, cough, dyspnea, rash, genital symptoms, or gastrointestinal complaints at presentation. He had no chronic medical conditions, took no regular medications, and reported no prior surgeries or known allergies. A detailed epidemiological exposure assessment was performed. The patient denied recent international travel, contact with individuals known to have Mpox or unexplained rash illnesses, healthcare-related exposure, attendance at mass gatherings, overnight stays in shared accommodation or hotels, occupational exposure to potentially infected individuals, recent domestic travel within the United Arab Emirates, and recognized sexual risk factors. Despite comprehensive exposure assessment, no definitive epidemiological source of infection was identified.

The patient was alert and hemodynamically stable. Temperature was 37.2°C, heart rate 102 beats per minute, blood pressure 120/91 mmHg, and oxygen saturation 99% on room air. The otolaryngology examination documented bilateral erythematous palatine tonsils with cryptic exudate and no abscess on direct visualization, an erythematous and edematous uvula without deviation, and erythematous pharyngeal mucosa. The oral mucosa was non-erythematous. There was no trismus, no stridor, no respiratory distress, and no drooling. Bilateral tender cervical lymphadenopathy was noted. The skin was documented as moist with no rash at the time of initial examination. Flexible laryngoscopy was not performed.

Laboratory studies on admission showed a leucocytosis of 14.0 × 10⁹/L (neutrophils 7.74 × 10⁹/L; lymphocytes 4.63 × 10⁹/L; monocytes 1.25 × 10⁹/L), platelet count 133 × 10⁹/L, and elevated CRP of 102 mg/L. A throat swab obtained at admission in the emergency department was sent for bacterial culture. Given the severity of the progressive odynophagia and inability to tolerate oral intake, CT of the neck was performed to assess for a possible deep neck complication, including an evolving peritonsillar abscess. CT of the neck demonstrated bulky, heterogeneous bilateral palatine tonsils with several ill-defined hypodense areas within and significant narrowing of the oropharyngeal lumen (Figure 1). Multiple enlarged cervical lymph nodes were noted, the largest measuring 14 mm at level III. The nasopharynx, epiglottis, aryepiglottic folds, vallecula, pyriform sinuses, vocal cords, laryngeal cartilages, and visualized trachea and esophagus were reported as normal. The radiologist’s impression was “features of tonsillitis with the possibility of evolving abscess.” No follow-up imaging was performed during the admission.

**Figure 1:**
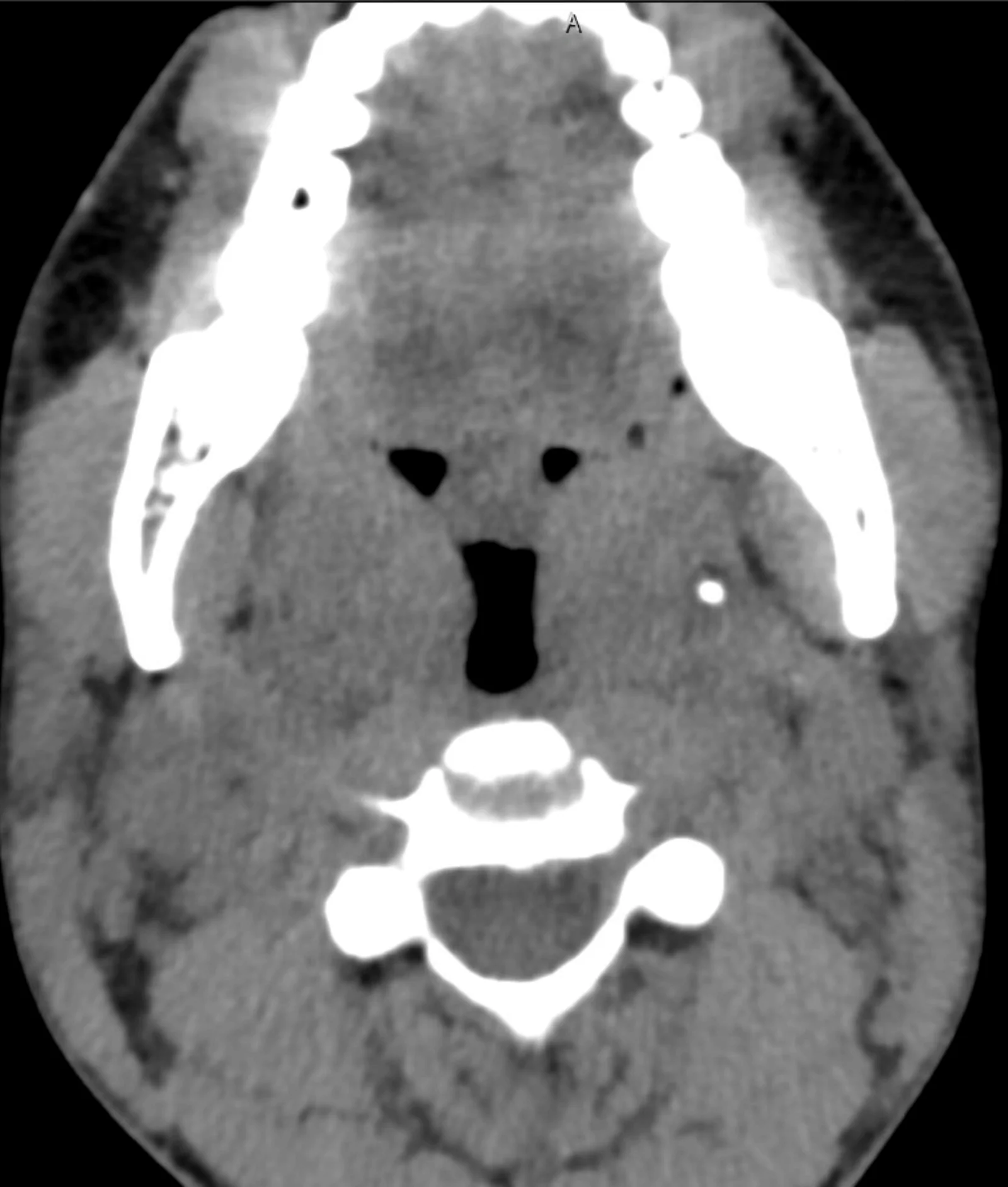
CT of the neck on admission An axial non-contrast CT image at the level of the oropharynx demonstrating bulky, heterogeneous bilateral palatine tonsils with ill-defined hypodense areas and significant narrowing of the oropharyngeal lumen. No discrete abscess is identified. Reactive cervical lymphadenopathy was noted on adjacent slices (not shown). The radiologist’s impression was “features of tonsillitis with the possibility of evolving abscess.”

The differential diagnosis included acute bacterial tonsillitis, evolving peritonsillar abscess, infectious mononucleosis (Epstein-Barr virus), cytomegalovirus infection, acute HIV seroconversion syndrome, herpes simplex virus infection, Coxsackievirus infection, gonococcal pharyngitis, and other viral causes of severe pharyngotonsillitis. These conditions were considered because of the patient’s prominent oropharyngeal symptoms, elevated inflammatory markers, and the absence of characteristic cutaneous lesions at presentation. The working diagnosis was acute bacterial tonsillitis with possible evolving peritonsillar abscess, and the patient was admitted under joint internal medicine and otolaryngology care. Empirical intravenous ceftriaxone 1 g twice daily and intravenous dexamethasone 8 mg daily were commenced, alongside intravenous fluid support and analgesia. Conservative airway monitoring was implemented; given the absence of stridor or respiratory distress, no airway intervention was indicated.

On hospital day 2, the patient developed mild, non-specific cutaneous lesions that did not initially suggest a defined diagnosis. On hospital day 3, the eruption progressed and became clinically characteristic: vesiculopustular lesions appeared on the face, trunk, back, upper limbs, and genital area, evolving sequentially through macular, papular, vesicular, and pustular stages, with several lesions showing central umbilication. Notably, lesions were also present on the palms and dorsa of the hands. The morphology and distribution prompted clinical suspicion of Mpox.

The patient was placed in single-room isolation with contact and droplet precautions. A lesion swab was obtained for orthopoxvirus and mpox virus testing. On hospital day 4, the throat swab culture was finalized and reported as the growth of normal upper respiratory tract flora only, with no beta-hemolytic streptococci isolated. On hospital day 6, HIV antigen/antibody combination screening returned negative. Epstein-Barr virus testing showed negative viral capsid antigen IgM, positive viral capsid antigen IgG and nuclear antigen IgG, and undetectable Epstein-Barr virus PCR. Although infectious mononucleosis was considered initially, the emergence of characteristic cutaneous lesions prompted targeted Mpox testing. On hospital day 7, real-time PCR on the skin lesion swab returned positive for the Mpox virus, with a cycle threshold value of 12, consistent with a high viral load. The qualitative assay targeted Mpox virus and other orthopoxviruses using specific primers and probes, with internal and external controls to ensure reliability and detect potential inhibition. Clade-specific identification and additional sexually transmitted infection screening were not performed at our facility. Tecovirimat was not administered because the patient demonstrated progressive clinical improvement with supportive management and local institutional protocols did not recommend antiviral therapy in this setting. Additional sexually transmitted infection screening beyond HIV was not performed because comprehensive sexually transmitted infection testing was not routinely available within our facility at the time of admission and the patient denied recognized sexual risk factors; nevertheless, coinfections with sexually transmitted infections have been reported in Mpox outbreak cohorts and therefore cannot be completely excluded.

The patient remained haemodynamically stable throughout admission, with no respiratory deterioration and no requirement for airway intervention. Tonsillar inflammation and oropharyngeal symptoms gradually improved with supportive care, and oral intake was progressively re-established. Cutaneous lesions evolved through pustular and crusting stages without secondary bacterial infection or other complications. Following laboratory confirmation of Mpox on hospital day 7, dedicated written informed consent for clinical photography and publication was obtained from the patient. Representative clinical photographs of the oropharyngeal (Figure 2) and cutaneous (Figure 3) lesions were subsequently obtained on hospital day 9, prior to transfer to the designated isolation facility on the same day. On hospital day 9, in line with national public health protocols, the patient was transferred to a designated Mpox isolation facility for ongoing care. He was discharged home on hospital day 27 in stable clinical condition, with complete resolution of pharyngeal symptoms and progressive healing of cutaneous lesions. Discharge instructions included continued isolation for several days, personal hygiene measures, and clear advice to return if symptoms recurred. A structured post-discharge telephone follow-up was completed on post-discharge day 1; the patient reported feeling well, with all lesions healed, no new lesions, and no recurrent symptoms. The total duration from initial admission to final discharge was 27 days, and no long-term complications were identified.

**Figure 2:**
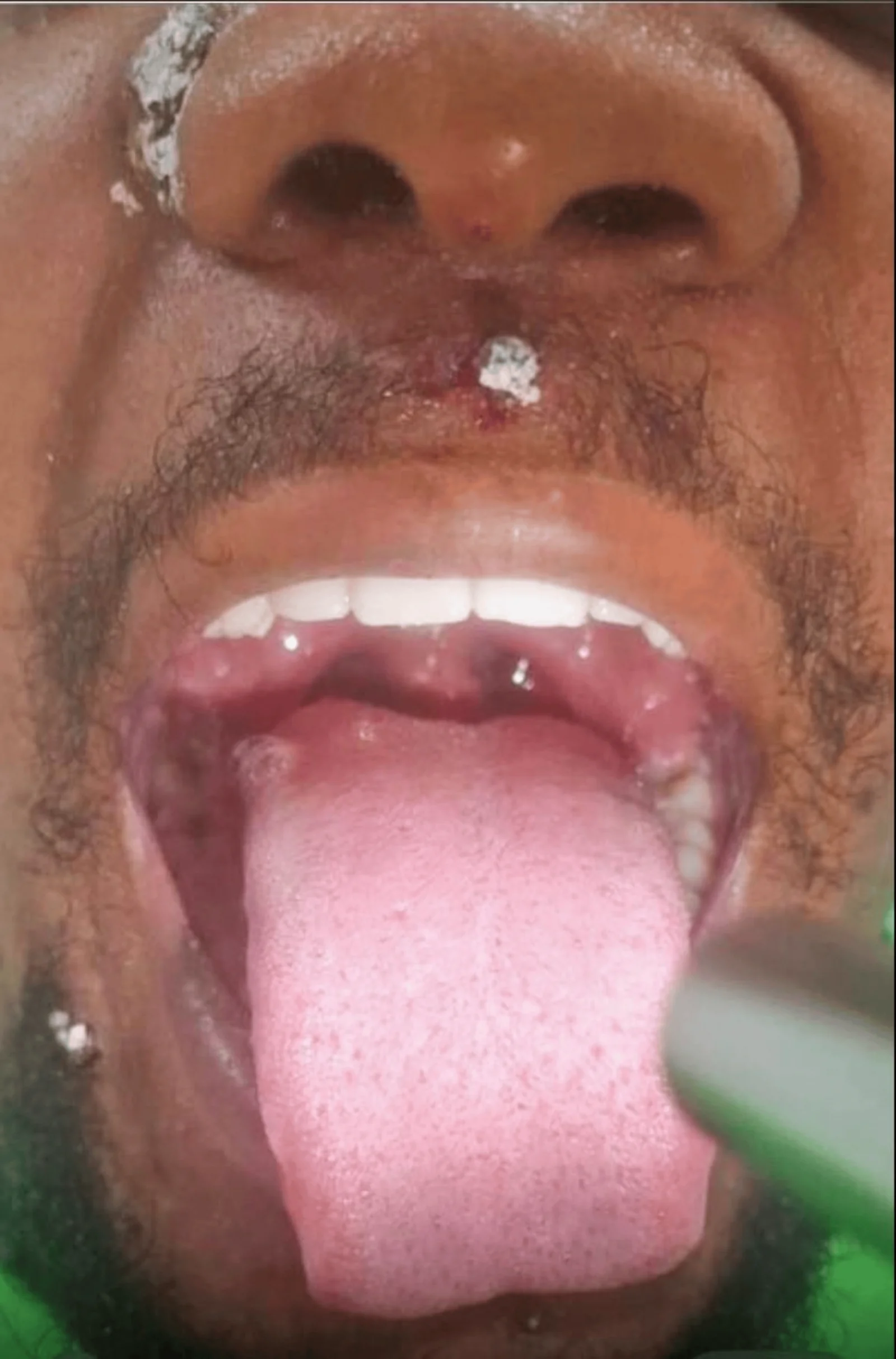
Oropharyngeal lesions Clinical photograph of the oropharynx obtained on hospital day 9, following laboratory confirmation of Mpox and after dedicated written informed consent for clinical photography and publication had been obtained from the patient. The image shows scattered ulcerative and vesiculopustular lesions on the soft palate, tonsillar pillars, and tongue base, accompanied by a small, crusted lesion on the upper cutaneous lip.

**Figure 3:**
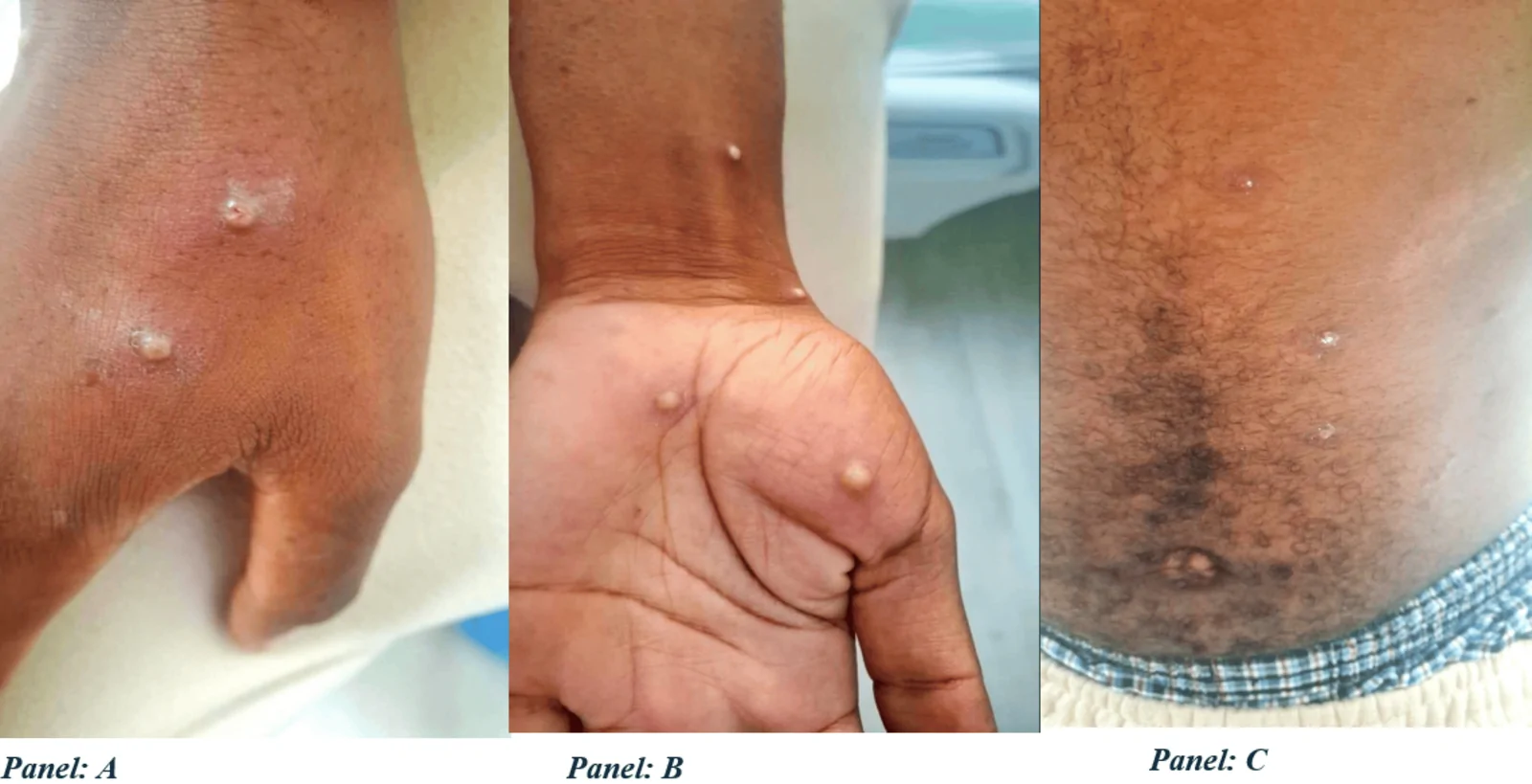
Cutaneous lesions Representative clinical photographs of cutaneous lesions obtained on hospital day 9, following laboratory confirmation of Mpox and after dedicated written informed consent for clinical photography and publication had been obtained from the patient. The images depict Mpox lesions in pustular and early crusting stages, consistent with the natural evolution of lesions approximately one week after their initial appearance. Panel A: dorsum of the hand with umbilicated pustular lesions; Panel B: palmar surface with firm pearly pustules, a recognised feature of Mpox; Panel C: trunk and lower abdomen showing scattered lesions in pustular and crusting stages, illustrating the polymorphic appearance characteristic of the current outbreak phenotype. All images were captured on the same day of illness (hospital day 9).

A formal written patient narrative could not be obtained because the patient had been transferred to a designated isolation facility and was no longer available for an in-person interview. During the documented post-discharge telephone follow-up, the patient reported feeling well, with complete healing of lesions, no new lesions, and no recurrent symptoms. The patient’s clinical course is summarized in Table 1.

**Table 1:** Clinical timeline summarising the patient’s course from symptom onset through hospital admission, diagnostic investigations, emergence of cutaneous lesions, laboratory confirmation of Mpox, transfer to the designated isolation facility, discharge, and post-discharge follow-up.

Day	Hospital day	Clinical event
–2	–	Onset of sore throat and odynophagia.
0	Day 1	Admission; Bilateral erythematous tonsils with cryptic exudate; oedematous uvula; cervical lymphadenopathy. WBC 14.0 × 10⁹/L; CRP 102 mg/L. CT of the neck: significant oropharyngeal narrowing; radiologist’s impression of tonsillitis with possibility of evolving abscess. IV ceftriaxone and dexamethasone commenced. Throat swab was obtained.
1	Day 2	Mild, non-specific cutaneous lesions observed.
2	Day 3	Rash becomes clinically characteristic: vesiculopustular lesions on face, trunk, back, limbs, palms, and genitalia. Mpox suspected. Single-room isolation initiated with contact and droplet precautions. Lesion swab sent for PCR.
3	Day 4	Throat culture finalised: normal upper respiratory tract flora only; no beta-haemolytic streptococci isolated.
5	Day 6	HIV antigen/antibody combination screening: negative.
6	Day 7	Mpox virus PCR positive on lesion swab; cycle threshold value 12. Supportive care continued; antibacterial therapy reviewed.
8	Day 9	Transfer to designated national Mpox isolation facility.
27	Day 27	Discharge home in stable clinical condition with complete resolution of pharyngeal symptoms and healed cutaneous lesions. Discharge education provided, including isolation advice and instructions to return if symptoms recurred.
28	Post-discharge day 1	Post-discharge telephone follow-up completed. Patient reported feeling well, all lesions healed, no new lesions, and no recurrent symptoms.

The patient’s laboratory, microbiological, and radiological investigations during the admission, including the timing of testing and key diagnostic findings, are summarised in Table 2.

**Table 2:** Summary of the patient’s laboratory, microbiological, and radiological investigations during admission, including the timing of testing and key diagnostic findings.

Investigation	Day collected/resulted	Result	Reference range
WBC count	Day 1	14.0 × 10⁹/L	4.0–11.0
Neutrophils	Day 1	7.74 × 10⁹/L	2.0–7.5
Lymphocytes	Day 1	4.63 × 10⁹/L	1.0–4.0
Monocytes	Day 1	1.25 × 10⁹/L	0.2–1.0
Platelets	Day 1	133 × 10⁹/L	150–400
CRP	Day 1	102 mg/L	<5
CT neck (non-contrast)	Day 1	Bulky heterogeneous bilateral palatine tonsils with hypodense foci; significant oropharyngeal narrowing; cervical lymphadenopathy (largest 14 mm, level III); larynx, trachea, and oesophagus normal. Radiologist’s impression: features of tonsillitis with possibility of evolving abscess.	–
Throat swab culture	Collected Day 1; resulted Day 4	Normal upper respiratory tract flora only; no beta-haemolytic streptococci isolated	–
HIV antigen/antibody combination	Day 6	Negative	Negative
Mpox/orthopoxvirus real-time PCR (lesion swab)	Day 7	Mpox virus detected; cycle threshold value 12	Not detected

## Discussion

Summary and clinical significance

We report a case of laboratory-confirmed Mpox in a previously healthy 28-year-old man in the United Arab Emirates who presented with severe acute tonsillitis, an edematous uvula, and CT evidence of significant oropharyngeal luminal narrowing. The radiologist’s impression on initial CT was “features of tonsillitis with possibility of evolving abscess,” reinforcing the working diagnosis of bacterial infection at the time of admission. The characteristic vesiculopustular eruption emerged only after admission and after initiation of empirical antibacterial therapy. The case illustrates two clinically important points: first, that Mpox can present as severe pharyngotonsillitis closely resembling acute bacterial infection in its initial phase; and second, that supportive radiographic findings may further entrench an incorrect bacterial working diagnosis. Documented telephone follow-up after discharge confirmed sustained clinical recovery with full lesion resolution and no recurrence of symptoms.

Atypical Mpox phenotypes in the post-2022 era

The clinical phenotype of Mpox has evolved substantially since the 2022 global outbreak. Whereas the classical course described in earlier African case series emphasised a febrile prodrome followed by a centrifugal vesiculopustular rash, the 2022 outbreak, driven predominantly by clade IIb, has been associated with a higher proportion of mucosal-first presentations, fewer total lesions, and frequent involvement of the anogenital and oropharyngeal regions [5,6]. Large case series have documented sore throat, odynophagia, and tonsillar lesions in a substantial subset of patients [7]. More recent reports describing clade Ib infection in central Africa have similarly demonstrated heterogeneous clinical patterns, although clade-specific data remain limited in many regional settings. In the United Arab Emirates and the wider Gulf region, sporadic Mpox cases have been reported, predominantly linked to international travel; published descriptions of locally relevant atypical presentations remain limited. Our case adds evidence from the United Arab Emirates to a growing body of literature recognising that the clinical gateway to Mpox may, in some patients, be the oropharynx rather than the skin or anogenital region.

Diagnostic delay in a low-risk, immunocompetent patient

The principal teaching point of this case concerns diagnostic delay in a patient whose epidemiological and clinical profile did not, on initial assessment, raise suspicion for Mpox. Our patient was previously healthy and denied recent travel, sexual contact, and known exposure to confirmed or suspected Mpox cases. In contrast, large outbreak cohorts since 2022 have predominantly described Mpox in men who have sex with men and in people living with HIV [5,6]. The initial clinical picture in our case, severe sore throat, bilateral tonsillar exudate, leucocytosis, and elevated CRP, corresponded closely to acute bacterial tonsillitis, and the CT impression of features compatible with tonsillitis and possible evolving abscess reinforced this working diagnosis. The throat swab obtained at admission, finalised on hospital day 4, yielded only normal upper respiratory tract flora with no beta-haemolytic streptococci, although by this time the cutaneous eruption had already raised the suspicion of Mpox. Although elevated inflammatory markers and exudative tonsillitis are widely associated with bacterial pharyngitis, both findings have been reported in Mpox with oropharyngeal involvement and do not reliably distinguish the two etiologies. In this patient, the cutaneous eruption was the eventual diagnostic clue: subtle, non-specific skin lesions on hospital day 2 progressed by day 3 into the characteristic vesiculopustular distribution that prompted Mpox testing. The take-home message for clinicians is twofold: severe pharyngotonsillitis with a negative bacterial culture, particularly when the clinical course does not follow that typically expected for bacterial infection, should prompt early consideration of viral aetiologies including Mpox; and Mpox should not be excluded on the basis of an apparently low-risk profile alone, since atypical presentations can occur in immunocompetent individuals without identifiable exposure.

Severe oropharyngeal inflammation without true airway compromise

A secondary feature of this case is the radiographic finding of significant oropharyngeal luminal narrowing in the absence of clinical airway compromise. The CT report described bulky, heterogeneous palatine tonsils with hypodense foci and significant narrowing of the oropharyngeal lumen, while the larynx, trachea, and supraglottic structures were reported as normal. Clinically, the patient maintained normal oxygen saturation throughout, with no stridor, drooling, or respiratory distress, and no airway intervention was required. Flexible laryngoscopy was not performed. This distinction between radiographic luminal narrowing and functional airway compromise is important: it argues against routine invasive airway management in similar cases while supporting early multidisciplinary involvement, including otolaryngology and anesthesia, for vigilant clinical monitoring. Severe oropharyngeal Mpox with airway involvement has been described elsewhere [8], including a previously reported case in which clinical airway compromise developed and was successfully managed with intravenous corticosteroids and tecovirimat [9]. Our case adds the observation that significant CT-confirmed oropharyngeal narrowing can occur without functional airway compromise, supporting an individualised, monitoring-based approach to airway management in Mpox-associated tonsillitis.

Although radiological findings raised concern for an evolving abscess, the absence of a well-defined rim-enhancing collection and the subsequent clinical course suggest that the observed changes were more likely attributable to severe Mpox-associated inflammatory involvement rather than true suppurative disease. Similar imaging appearances have been described in isolated reports of oropharyngeal Mpox, although detailed radiological characterisation remains limited in the literature.

Pre-diagnostic corticosteroid exposure

Our patient received intravenous dexamethasone for two days before Mpox was suspected, in keeping with standard practice for severe pharyngeal inflammation. Although corticosteroids are theoretically of concern in undiagnosed viral infections through their immunomodulatory effects, the limited evidence available does not suggest harm in short-course Mpox exposure, and our patient recovered fully without complication. This case neither demonstrates safety nor establishes risk; rather, it reinforces the argument for earlier diagnostic consideration of Mpox to permit informed treatment decisions.

Infection control implications

From an infection control perspective, the six-day interval between presentation and laboratory confirmation represents a window of potential nosocomial exposure. Close-contact and potentially aerosol-generating procedures performed during this period, including oropharyngeal examination, CT scanning, and routine nursing care, carry transmission risk in undiagnosed Mpox [10]. In endemic and non-endemic regions alike, low-threshold isolation when severe atypical pharyngotonsillitis presents with mucosal edema and prominent cervical lymphadenopathy may reduce unnecessary exposure to healthcare workers and other patients, even before Mpox is confirmed.

Limitations

This report has several limitations. As a single-case observation, the findings are illustrative rather than generalisable. Clade-specific identification and quantitative viral load were not available at our institution; the qualitative real-time PCR confirmed Mpox virus infection, and the cycle threshold value of 12 indicated a high viral load, but genomic characterisation could not be performed. The patient denied recognised risk factors, including travel, sexual contact, and known exposure to confirmed cases; while this is documented honestly, undisclosed exposure cannot be excluded. No further sexually transmitted infection screening was undertaken at our facility. Flexible laryngoscopy was not performed; documented oropharyngeal findings derive from direct otolaryngology examination and CT imaging. As detailed in the Case Presentation section, antiviral therapy with tecovirimat was not administered in view of progressive clinical improvement with supportive management and local institutional protocols. Post-discharge follow-up consisted of a single structured telephone contact on the day following discharge, confirming clinical recovery; longer-term follow-up data are not available.

Learning points

Several practical lessons emerge from this case. Mpox may initially present as severe exudative pharyngotonsillitis before the appearance of characteristic cutaneous lesions, and CT-confirmed oropharyngeal narrowing may occur without functional airway compromise or the need for airway intervention. Severe pharyngotonsillitis accompanied by negative bacterial cultures, prominent cervical lymphadenopathy, or evolving skin lesions should therefore prompt consideration of Mpox. Diagnostic delay may occur even in immunocompetent patients without recognised epidemiological risk factors. Early isolation may reduce healthcare-associated transmission and unnecessary antibacterial exposure. Finally, radiological findings suggestive of an evolving tonsillar abscess do not exclude Mpox and should be interpreted in conjunction with clinical progression and microbiological findings.

## Conclusions

This case demonstrates that Mpox can present initially as severe acute tonsillitis with bilateral exudative inflammation, uvular edema, and CT evidence of significant oropharyngeal luminal narrowing closely resembling acute bacterial tonsillitis with possible evolving abscess in a previously healthy, young man without recognised epidemiological risk factors. The characteristic cutaneous eruption may emerge only after admission and after initial antibacterial therapy, contributing to diagnostic delay. Clinicians evaluating patients with severe pharyngotonsillitis, particularly when bacterial cultures are negative or when even subtle cutaneous changes appear during admission, should consider Mpox within the differential diagnosis regardless of perceived epidemiological risk. Early recognition enables timely isolation, reduces unnecessary antibacterial exposure, prevents potential nosocomial transmission, and protects healthcare workers. This case expands the growing body of evidence that Mpox may initially manifest as isolated oropharyngeal disease before the appearance of characteristic cutaneous findings.

As Mpox continues to circulate globally with evolving clinical phenotypes, awareness of atypical presentations remains essential across emergency, internal medicine, and otolaryngology services in the United Arab Emirates and elsewhere.
